# Positive and Negative Affect Schedule (PANAS): psychometric properties of the online Spanish version in a clinical sample with emotional disorders

**DOI:** 10.1186/s12888-020-2472-1

**Published:** 2020-02-10

**Authors:** Amanda Díaz-García, Alberto González-Robles, Sonia Mor, Adriana Mira, Soledad Quero, Azucena García-Palacios, Rosa María Baños, Cristina Botella

**Affiliations:** 1 Department of Basic and Clinical Psychology, and Psychobiology, Universitat Jaume I, Castellón, Spain; 2 Department of Personality, Evaluation and Psychological Treatments, Universidad de Valencia, Valencia, Spain; 3 CIBER Fisiopatología Obesidad y Nutrición (CIBERObn), Instituto Carlos III, Madrid, Spain

**Keywords:** Positive and negative affect, Validation, Assessment, Psychometrics, Online, Emotional disorders

## Abstract

**Background:**

The Positive and Negative Affect Schedule (PANAS) is the most widely and frequently used scale to assess positive and negative affect. The PANAS has been validated in several languages, and it has shown excellent psychometric properties in the general population and some clinical samples, such as forensic samples, substance users, and adult women with fibromyalgia. Nevertheless, the psychometric properties of the scale have not yet been examined in clinical samples with anxiety, depressive, and adjustment disorders. In addition, the proliferation of Internet-based treatments has led to the development of a wide range of assessments conducted online with digital versions of pen and paper self-report questionnaires. However, no validations have been carried out to analyze the psychometric properties of the online version of the PANAS. The present study investigates the psychometric properties of the online Spanish version of the PANAS in a clinical sample of individuals with emotional disorders.

**Methods:**

The sample was composed of 595 Spanish adult volunteers with a diagnosis of depressive disorder (*n* = 237), anxiety disorder (*n* = 284), or adjustment disorder (*n* = 74). Factor structure, construct validity, internal consistency, and sensitivity to change were analyzed.

**Results:**

Confirmatory factor analysis yielded a latent structure of two independent factors, consistent with previous validations of the instrument. The analyses showed adequate convergent and discriminant validity, good internal consistency as well as sensitivity to change.

**Conclusions:**

Overall, the results obtained in this study show that the online version of the PANAS has adequate psychometric properties for the assessment of positive and negative affect in a Spanish clinical population.

## Introduction

The study of the structure of affect has been particularly important in increasing psychopathological and clinical knowledge about mental disorders. Affect plays a central role in human experience [1], and the term has been used to refer to anything emotional, that is, feelings, preferences, emotions, moods, and affective traits [2, 3]. Affect is regarded as a psychological construct that refers to mental states involving evaluative feelings (e.g., feeling good-bad, liking-disliking a situation) [4]. Nevertheless, the difficulty of achieving reliable measurements and the absence of a unified model of affective processes have limited its scientific study [5].

In recent decades, a growing number of studies have tried to investigate the structure of affect. Most of these studies agree that the affective experience has two dominant dimensions, namely, positive affect (PA) and negative affect (NA) [6–8]. Based on the pioneering work by Bradburn [9], PA and NA have been described as two independent unipolar dimensions of affect that include all the affective states with a positive valence (joy, enthusiasm, crush, etc.) or a negative valence (anger, fear, anxiety, etc.). Furthermore, in a re-analysis of a large number of studies on affect, Watson and Tellegen (1985) concluded that the two main factors that appeared consistently were positive and negative affect, and they presented the consensual two-factor model. These two factors have been conceptualized as two independent and uncorrelated dimensions of affect [10]. Whereas PA reflects the “extent to which individuals feel enthusiastic, active, and alert” [11], NA involves a variety of aversive mood states, such as anger, guilt, and fear. The data suggest that low levels of PA are related to and predict the onset of depression [12], and that high PA is associated with greater well-being [13]. By contrast, low NA indicates a state of calmness and serenity, whereas high NA is characteristic of anxiety [11]. The two dimensions of affect (PA and NA) have been crucial in the conceptual differentiation between depression and anxiety disorders [14]. In addition, PA and NA have also been strongly related to Extraversion and Neuroticism personality dimensions, respectively [15].

It is important to highlight that, without an adequate and reliable measurement of affect, it is not feasible to conduct research that provides empirical support in this field. Thus, the Positive and Negative Affect Schedule (PANAS) was developed for this purpose. The PANAS is the most widely and frequently used scale to assess PA and NA. The original PANAS scale was designed by Watson et al. (1988) as a brief and easy to administer measure to assess positive and negative affect and, thus, obtain affect descriptors that are as pure as possible. In this regard, the authors finally isolated 10 PA markers (PA subscale) and 10 NA markers (NA subscale), which are the 20 items on the current version of the PANAS. On the one hand, the PA subscale reflects the extent to which a person feels interested, excited, strong, enthusiastic, proud, alert, inspired, determined, attentive, and active. On the other hand, the NA subscale includes descriptors such as stressed, upset, guilty, scared, hostile, irritable, ashamed, nervous, jittery, and afraid. All the items are rated on a scale ranging from 1 (“very slightly or not at all”) to 5 (“extremely”).

Although the PANAS was initially designed and developed in North America [11], it has been validated in several languages in both western (e.g. Italy: [16]; France and Canada: [17]; Hungary: [18]) and non-western countries (e.g. Turkey: [19]; Mexico: [20]; Korea: [21]; Argentina: [22]; Brasil: [23]; Africa: [24]; India: [25]; Pakistan: [26]). The results of these studies have shown excellent internal consistency and discriminant validity. In Spain, the PANAS has also demonstrated high internal consistency (alpha = .89 and .91 for PA and NA in women, respectively, and alpha = .87 and .89 for PA and NA in men, respectively) in college students [27]. Nevertheless, the PANAS has only been validated in the general population, both its original version [11] and the Spanish version [27], as well as in the other previously mentioned countries. However, some validations have been carried out with clinical samples, such as forensic samples [28], psychiatric outpatients [21], substance users [29], and adult women with fibromyalgia [30].

Currently, the proliferation of Internet-based treatments has led to the development of a wide range of assessments conducted online using digital versions of pen and paper self-report questionnaires [31, 32]. However, although paper and online versions of the same instrument correlate strongly, they may differ in their psychometrics properties [31]. Therefore, the need for validated assessments administered online is increasingly evident [33], reinforcing the importance of evidence-based assessment, that is, the use of research and theory in the selection of assessment targets, the methods and measures used in the assessment, and the assessment process itself [34]. Given the key role of assessment in evidence-based practice, the development and promotion of rigorous assessment instruments is crucial for mental health researchers and clinicians. Thus, the validation of online scales can have a direct impact on the availability of evidence-based assessments because they can be administered more extensively.

Although the PANAS has been validated in the general population, the psychometric properties of this scale have not yet been examined in clinical samples with anxiety, depressive, and adjustment disorders. In addition, to the best of our knowledge, no validations have been carried out to analyze the psychometric properties of the online version of the scale. Therefore, this is the first study to evaluate the psychometric properties of the PANAS administered online in a Spanish clinical population.

### Current study

The present study investigates the psychometric properties of the online Spanish version of the PANAS in a clinical sample of individuals with depressive, anxiety, and adjustment disorders. The main objectives of this study is the translation and validation of the scale, with the specific objectives of: a) examining the scale’s internal consistency; b) assessing the construct validity; c) determining the factor structure of the scale; and d) analyzing its sensitivity to change.

## Method

### Spanish translation of the PANAS

The goal of translation is to achieve equivalence between the original and the translated version of the scale. The PANAS was translated into Spanish in order to assess its psychometric properties in this population. First, two independent translations were developed. A native Spanish-speaking translator with knowledge of psychology and emotional disorders translated the PANAS items from English to Spanish. Second, a Spanish-English bilingual speaker who was not familiar with the questionnaire translated the instrument from Spanish to English. Then, the scale was translated back again into the source language of the independent translators in a “back-translation” procedure. Finally, a committee integrated by the senior supervisors of the study looked at the original and the back-translated items of the scale and resolved discrepancies. The Spanish version of the PANAS was found to be an accurate translation of the original English version (see Additional file 1 for Spanish version of the PANAS).

### Participants and procedure

Participants were recruited from patients attending the Emotional Disorders Clinic at Universitat Jaume I (Castellon, Spain). Three samples of participants who were waiting to receive an online psychological treatment took part in the current study. Sample 1 included participants with a diagnosis of anxiety and/or depressive disorder that received a transdiagnostic Internet-based protocol for emotional disorders; sample 2 included participants with a diagnosis of adjustment disorder that participated in an Internet-based protocol for the treatment of these disorders; and sample 3 included participants with a diagnosis of depression that received a brief online depression protocol. A full description of the treatments can be found elsewhere [35–37]. All the participants were assessed with a structured diagnostic interview. The interviews lasted approximately an hour and were conducted by pre-doctoral students who had been fully trained for this purpose. Once the inclusion criteria had been confirmed (see “Inclusion and exclusion criteria” section below), participants signed an online informed consent, and the anonymity and confidentiality of the data were guaranteed. Before participants could access the treatment modules, their data were collected using an online battery of questionnaires (described below in greater detail in the Measures section) administered over the Internet through a web platform (https://www.psicologiaytecnologia.com/) designed by our research group. Part of the sample completed the PANAS before and immediately after receiving the Internet-based treatment. All the research projects in which the sample participated had been approved by the Ethics Committee at Universitat Jaume I.

### Inclusion and exclusion criteria

Inclusion to the study required the presence of at least one diagnosis of depressive, anxiety or adjustment disorders. Participants also had to be 18 years or older, be fluent in understanding and reading Spanish, have access to Internet at home and an email address, and give online informed consent. Participants with a severe mental disorder (schizophrenia, bipolar disorder, and alcohol and/or substance dependence disorder) or high risk of suicide were excluded from the study.

### Measures

#### Screening measures

***Mini International Neuropsychiatric Interview*** [38]. The MINI is a short, structured, diagnostic interview for DSM-IV and ICD- diagnoses. This interview has excellent test-retest and inter-rater reliability (k = .88–1.00), as well as adequate concurrent validity with the Composite International Diagnostic Interview [39]. The Spanish validation was used in this study [40].

***Diagnostic Interview for Adjustment Disorders*** (Rachyla, Botella, Mor, Tur, Lopez-Montoyo, Baños & Quero, in preparation). This measure is a semi-structured interview based on the ICD-10, DSM-IV-TR, and Structured Clinical Interview for the DSM-IV (SCID-CV) for the diagnosis of anxiety disorders. It assesses the number of stressful events in the person’s life in the past few months and their related symptomatology. This interview is currently undergoing a validation process.

#### Validation measures

***Positive and Negative Affect Schedule-Trait*** (PANAS [11];. The questionnaire contains 20 items on two subscales that assess a person’s positive and negative trait affect using a 5-point scale (1= “very slightly or not at all”; 5=” extremely”). Both the original validation [11] and the Spanish validation [27] showed two clearly differentiated factors (PA and NA) and good psychometric properties. In the current study, Cronbach alpha was excellent for the PANAS-P (.91) and good for the PANAS-N (.87).

***Beck Depression Inventory****(BDI-II* [41];, using the Spanish adaptation [42]). The questionnaire includes 21 items that assess depression severity using four response options ranging from 0 to 3. The instrument shows adequate internal consistency (Cronbach alpha ranging from .76 to .96) and good test-retest reliability (0.80). The psychometric properties of the Spanish adaptation also show high internal consistency: Cronbach alpha of .87 in the general population [43] and .89 in a clinical population [44]. Cronbach alpha for the BDI-II in this study was excellent (.91).

***Beck Anxiety Inventory****(BAI* [45];, using the Spanish adaptation [46]. The questionnaire contains 21 items that assess anxiety using a four-point Likert scale. The Spanish adaptation showed high internal consistency (α = .93) [47]. In the present study, Cronbach alpha for the BAI was excellent (.91).

***Overall Anxiety Severity and Impairment Scale*** (*OASIS* [48];, using the Spanish version [46]. This questionnaire assesses the frequency and severity of anxiety through five items rated on a 0 to 4-point scale. Cronbach alpha for the scale was .80, and it showed good test-retest reliability (k = 5, .82). The Spanish version of the OASIS has shown good internal consistency (α = .86), and convergent and discriminant validity [49]. Cronbach alpha for the OASIS in the current study was good (.87).

***Patient Health Questionnaire-9*** (PHQ-9 [50];. This short 9-item questionnaire (responses ranging from 0 to 3) assesses depressive symptomatology in the previous two weeks. Cronbach alpha for the questionnaire was .89, and test-retest reliability was also excellent [50]. The Spanish validation of the PHQ-9 has demonstrated a good internal consistency in a sample of primary care patients (α = .89) [51]. Cronbach alpha for the PHQ-9 in this study was good (.85).

***NEO-five factor Inventory*** [52]. The NEO FFI, the short version of the NEO-PI-R, assesses five personality dimensions through 60 items rated on a Likert scale ranging from 0 to 4. The present study used the Neuroticism and Extraversion scales, each containing 12 items. Internal consistency of the Extraversion subscale was .82, and .84 for the Neuroticism scale. Test-retest reliability was .86 for Extraversion and .89 for Neuroticism. The Spanish version of the questionnaire also showed good properties [53]. In the present study, Cronbach alpha was good for both the Neuroticism (.80) and Extraversion (.86) subscales.

### Data analysis

Reliability was analyzed by calculating internal consistency (Cronbach alpha) separately with the items on the PANAS-P and the PANAS-N.

In order to analyze the latent structure of the PANAS, Confirmatory Factor Analysis (CFA) was performed because the PANAS has previously been validated, with a theoretical structure of two correlated factors repeatedly found [54]. WLSMV (Weighted Least Square Mean and Variance corrected) estimation was employed because the variables did not fulfill multivariate normality, and the items were categorical (i.e. Likert-type items). WLSMV is one of the best estimation methods for this type of data [55, 56]. Model fit was analyzed using several indices from different families, as recommended in the literature [57, 58]. Specifically, all the indices and statistics used with WLSMV are presented: a) chi-square; b) Comparative Fit Index (CFI); Standardized Root Mean Residual (SRMR); and c) Root Mean Square Error of Approximation (RMSEA). Criteria for acceptable model fit were CFI above .90 (preferably above .95) and RMSEA and/or SRMR below .08 [59]. CFA was estimated with Mplus v.8 [60].

To examine construct validity, correlations between the PANAS-P and PANAS-N and measures of anxiety (BAI, OASIS), depression (BDI-II, PHQ-9), and temperament (NEO-FFI) were calculated. Following Cohen’s benchmarks [61], correlation values were interpreted as follows: effect sizes between .10 and .30 would be considered small, between .30 and .50 would be medium, and .50 or above would be large. A negative but medium correlation between PANAS-P and PANAS-N was expected. A negative and medium correlation was expected between PANAS-P and the anxiety (BAI, OASIS) and depression measures (BDI-II, PHQ-9). By contrast, a positive but medium correlation was expected between PANAS-N and the anxiety and depression measures. Additionally, we hypothesized positive medium correlations between PANAS-P and the NEO-FFI-E, and between PANAS-N and the NEO-FFI-N. Finally, negative but medium correlations were expected between PANAS-P and the NEO-FFI-N, and between PANAS-N and the NEO-FFI-E. This prediction was theoretically based on the commonalities between the affect constructs and the personality dimensions of neuroticism and extraversion [62, 63].

One-way ANOVAs were performed to analyze whether there were statistically significant differences in the scores on the two PANAS subscales (PANAS-P and PANAS-N) based on gender, marital status, education, principal diagnosis, and number of comorbid diagnoses. Correlations between age and the PANAS subscales were calculated to find any associations between these variables. Additionally, reliability was analyzed by calculating internal consistency (Cronbach alpha). These analyses were performed separately with the items on the PANAS-P and the PANAS-N.

Finally, to analyze the sensitivity of the PANAS-P and PANAS-N scores to change, means and standard deviations for the pretest and posttest were calculated for the three samples. For each sample, minimum and maximum PANAS-P and PANAS-N scores were obtained from the treatment pretest and control groups to check for potential floor or ceiling effects. Evidence of floor or ceiling effects is present when more than 15% of the participants obtain the lowest or highest possible score on the test, respectively (in our case, 10 and 50; cf. [64]. In addition, for each sample, *t*-tests were applied to test the statistical significance of the pretest-posttest mean differences in the treatment group. Furthermore, to quantify the sensitivity of the PANAS-P and PANAS-N scores to change, the standardized mean change index was used as the effect size, defined as the difference between the pretest and posttest means, divided by the standard deviation of the change scores in the treatment group:

$$ d=c(m){\overline{y}}_{Pre}-{\overline{y}}_{Post}/{SD}_{Change} $$

with ${\overline{\mathrm{y}}}_{\mathrm{Pre}}$ and ${\overline{\mathrm{y}}}_{\mathrm{Post}}$ being the pretest and posttest means of the treatment group. The positive bias of the *d* index for small sample sizes was corrected with the *c*(*m*) correction factor:

$$ c(m)=1-3/\left(4N-5\right) $$

In addition, 95% confidence intervals for the *d* indices were calculated by means of *d* ± 1.96x*SE*(*d*), with 1.96 being the 97.5 percentile of the standard normal distribution, and *SE*(*d*) being the standard error of the *d* index [65]:

$$ SE(d)=\sqrt{c{(m)}^2\mathrm{x}\left(1/n\right)\mathrm{x}\left(n-1/n-3\right)\mathrm{x}\left(1+n{d}^2\right)-{d}^2} $$

All of these calculations were applied separately for studies 1 to 3. To offer a contextualized interpretation of the *d* indices, we used the results from a systematic review of meta-analyses on the efficacy of psychological treatments, which applied the standardized mean change index as the effect size [66]. In this review, percentiles 25, 50, and 75 of the *d* indices’ distribution were 0.64, 0.75, and 1.26, respectively. Therefore, a reasonable interpretation of these three values is that they reflect low, moderate, and large effect sizes.

## Results

### Sample description

The total sample was composed of 595 Spanish adult volunteers (195 men; 400 women), with a mean age of 37.38 years (SD = 12.54; range: 18–76). Most of the participants were married or living with a partner (*n* = 273; 45.89), and most had high-level studies (*n* = 352; 59.2%).

The patients had been diagnosed with depressive disorder (i.e. major depressive disorder, dysthymic disorder, depression not otherwise specified) (*n* = 237), anxiety disorder (i.e. generalized anxiety disorder, panic disorder/agoraphobia, social anxiety disorder, obsessive-compulsive disorder) (*n* = 284), and adjustment disorder (*n* = 74). In addition, 35% of the total sample presented at least one comorbid anxiety or depressive disorder. Table 1 presents a detailed description of the participants’ sociodemographic and clinical data.

**Table 1:** Sociodemographic and clinical characteristics of the total sample

Age in years, Mean (SD)	37.35 (12.55)
Sex, n (%)	
Female	400 (67.2)
Male	195 (32.8)
Relationship status, n (%)	
Single	261 (43.9)
Married/Partnered	273 (45.9)
Divorced	56 (9.4)
Widowed	5 (0.8)
Education level, n (%)	
No studies	2 (0.3)
Basic	79 (13.3)
Medium	162 (27.2)
High-level	352 (59.2)
Diagnosis category, n (%)	
Depression	237 (39.8)
Anxiety	284 (47.7)
Adjustment disorder	74 (12.4)
Principal diagnosis, n (%)	
MDD	222 (37.3)
DD	11 (1.8)
GAD	121 (20.3)
PD/AG	32 (5.4)
PD	18 (3)
AG	19 (3.2)
SAD	65 (10.9)
OCD	17 (2.9)
Anxiety NOS	12 (2)
Depression NOS	4 (0.7)
Adjustment disorder	74 (12.4)
Number of comorbid disorders	
0	385 (64.87)
1	147 (24.7)
2	36 (6.1)
≥ 3	26 (4.4)
Symptom severity, Mean (SD)	
PANAS-P	20.19 (6.91)
PANAS-N	29.07 (8.14)
BDI-II	25.41 (11.85)
PHQ-9	14.44 (6.06)
BAI	21.25 (11.50)
OASIS	9.55 (4.31)
NEO-FFI-N	31.57 (7.56)
NEO-FFI-E	21.96 (8.66)

*MDD* Major Depressive Disorder, *DD* Dysthymic Disorder, *GAD* Generalized Anxiety Disorder, *PD/AG* Panic Disorder/Agoraphobia, *SAD* Social Anxiety Disorder, *OCD* Obsessive-Compulsive Disorder, *Anxiety NOS* Anxiety Not Otherwise Specified, *Depression NOS* Depression Not Otherwise Specified, *PANAS-P* Positive and Negative Affect Schedule - Positive Affect, *PANAS-N* Positive and Negative Affect Schedule - Negative Affect, *BDI-II* Beck Depression Inventory, *PHQ-9* Patient Health Questionnaire, *BAI* Beck Anxiety Inventory, *OASIS* Overall Anxiety Severity and Impairment Scale, *NEO-FFI-N* Neo Five-Factor Inventory - Neuroticism; *NEO-FFI-E* NEO Five-Factor Inventory – Extraversion

### Reliability

#### Internal consistency

Cronbach alpha was .91 for the PANAS-P and .87 for the PANAS-N. Tables 2 and 3 display Cronbach alphas when omitting items, corrected correlations between each item and the total score, and correlations between the items on PANAS-P and PANAS-N, respectively.

**Table 2:** Cronbach alpha if item is deleted, corrected item-total score correlations, and correlations between items (PANAS-P)

			Correlations between items									
	Cronbach alpha if item deleted	Corrected item-total correlation	Item 1	Item 3	Item 5	Item 9	Item 10	Item 12	Item 14	Item 16	Item 17	Item 19
Item 1	.904	.717	1	.629	.600	.637	.463	.477	.533	.485	.477	.552
Item 3	.904	.718		1	.591	.750	.520	.444	.529	.437	.403	.556
Item 5	.902	.748			1	.658	.458	.595	.501	.498	.459	.681
Item 9	.901	.777				1	.538	.498	.586	.501	.438	.596
Item 10	.910	.606					1	.377	.492	.473	.382	.459
Item 12	.908	.645						1	.445	.467	.510	.594
Item 14	.907	.669							1	.528	.429	.515
Item 16	.908	.643								1	.489	.524
Item 17	.911	.593									1	.496
Item 19	.903	.735										1

All correlations were statistically significant at *p* < .01 (two-tailed); Item 1: Interested; Item 3: Excited; Item 5: Strong; Item 9: Enthusiastic; Item 10: Proud; Item 12: Alert; Item 14: Inspired; Item 16: Determined; Item 17: Attentive; Item 19: Active

**Table 3:** Cronbach alpha if item is deleted, corrected item-total score correlations, and correlations between items (PANAS-N)

			Correlations between items									
	Cronbach alpha if item deleted	Corrected item-total correlation	Item 2	Item 4	Item 6	Item 7	Item 8	Item 11	Item 13	Item 15	Item 18	Item 20
Item 2	.858	.576	1	.467	.318	.339	.368	.471	.243	.588	.528	.305
Item 4	.855	.607		1	.386	.354	.517	.612	.330	.391	.406	.317
Item 6	.860	.554			1	.452	.363	.333	.536	.353	.359	.311
Item 7	.852	.641				1	.308	.271	.428	.462	.461	.769
Item 8	.860	.545					1	.660	.279	.322	.324	.273
Item 11	.857	.574						1	.276	.398	.361	.259
Item 13	.863	.517							1	.331	.317	.423
Item 15	.853	.641								1	.686	.437
Item 18	.853	.640									1	.504
Item 20	.857	.591										1

All correlations were statistically significant at *p* < .01 (two-tailed); Item 2: Distressed; Item 4: Upset; Item 6: Guilty; Item 7: Scared; Item 8: Hostile; Item 11: Irritable; Item 13: Ashamed; Item 15: Nervous; Item 18: Jittery; Item 20: Afraid

#### Convergent and divergent validity

Table 4 shows the correlations between PANAS-P and PANAS-N and convergent and divergent validity measures. Significant correlations were found between both PANAS-P and PANAS-N and all the measures. As expected, a negative but medium correlation was found between PANAS-P and PANAS-N (*r* = −.30, *p* < .01). Negative and large correlations were found between PANAS-P and depression measures (BDI-II: *r* = −.56, *p* < .01; PHQ-9: *r* = −.52, *p* < .01), whereas the correlations between PANAS-P and anxiety measures were small to medium (BAI: *r* = −.23, *p* < .01; OASIS: *r* = −.39, *p* < .01). By contrast, positive large correlations were found between PANAS-N and depression (BDI-II: *r* = .63, *p* < .01; PHQ-9: *r* = .54, *p* < .01) and anxiety measures (BAI: *r* = .58, *p* < .01; OASIS: *r* = .64, *p* < .01). Finally, a negative but medium correlation was found between PANAS-P and NEO-FFI-N (*r* = −.39, *p* < .01), and a positive and medium correlation between PANAS-P and NEO-FFI-E (*r* = .44, *p* < .01). In addition, a positive and large correlation was found between PANAS-N and NEO-FFI-N (*r* = .65, *p* < .01), and a negative but small correlation between PANAS-N and NEO-FFI-E (*r* = −.24, *p* < .01).

**Table 4:** Correlations of PANAS-P and PANAS-N with convergent and divergent validity measures

	PANAS-P	PANAS-N	BDI-II	PHQ-9	BAI	OASIS	NEO-FFI-N	NEO-FFI-E
PANAS-P	1	−.30	−.56	−.52	−.23	−.39	−.39	.44
PANAS-N		1	.63	.54	.58	.64	.65	−.24
BDI-II			1	.83	.51	.66	.64	−.28
PHQ-9				1	.52	.63	.60	−.25
BAI					1	.63	.48	−.17
OASIS						1	.51	−.26
NEO-FFI-N							1	−.30
NEO-FFI-E								1

All correlations were statistically significant at *p* < .01 (two-tailed). *PANAS-P* Positive and Negative Affect Schedule - Positive Affect, *PANAS-N* Positive and Negative Affect Schedule - Negative Affect, *BDI-II* Beck Depression Inventory, *PHQ-9* Patient Health Questionnaire, *BAI* Beck Anxiety Inventory, *OASIS* Overall Anxiety Severity and Impairment Scale, *NEO-FFI-N* Neo Five-Factor Inventory – Neuroticism, *NEO-FFI-E* NEO Five-Factor Inventory – Extraversion

#### Confirmatory factor analysis

**Fig. 1:**
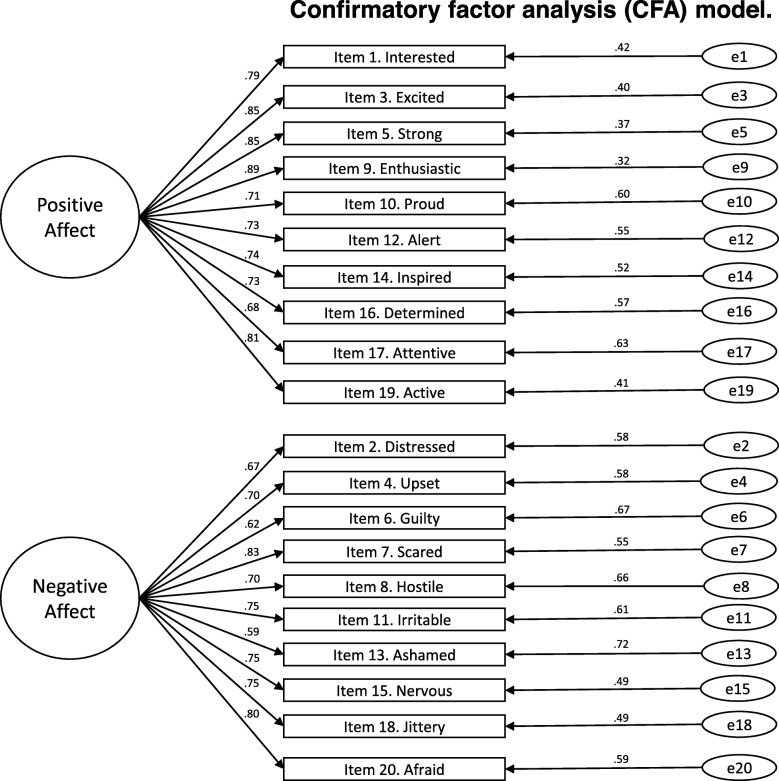
Confirmatory factor analysis (CFA) model. Note. Rectangles are measured variables, the large circles are the latent construct, and ellipses are residual variances. Factor loadings are standardized

The PANAS has a two-correlated factor structure, positive and negative affect. We tested this two-factor structure with a confirmatory factor analysis. The model reasonably fitted the observed data: χ^2^(169) = 1425.31, *p* < .001, χ^2^(169) = 8.43, RMSEA = .112 CI [.106–.117], CFI = .917, TLI = .907, SRMR = .076. Fit was adequate according to both the CFI and the SRMR. RMSEA was a little higher than expected. However, and taking into account that the parameter estimates were all statistically significant and very large, we can conclude that the model has an adequate fit. Figure 1 shows the CFA model. As mentioned above, all factor loadings were statistically significant (*p* < .001) and large. With regard to the first factor (positive affect), all standardized loadings were in the range from .68 to .85. Regarding the second factor (negative affect), again standardized loadings were large, with a minimum of .59 and a maximum of .83.

#### PANAS and sociodemographic and clinical variables

In the total sample, the mean PANAS-P score was 20.19 (SD = 6.91), and the mean PANAS-N score was 29.07 (SD = 8.14). Tables 5 and 6 show the means and standard deviations for each item and the total score on both the PANAS-P and the PANAS-N by diagnosis category.

**Table 5:** Descriptive statistics for each item and the total score on the PANAS-P

	Depression (*n* = 237)		Anxiety (*n* = 284)		Adjustment disorder (*n* = 74)	
	M	SD	M	SD	M	SD
Item 1	2.24	.94	2.54	1.01	2.51	.98
Item 3	1.77	.85	2.19	.99	2.08	1.02
Item 5	1.80	.83	2.07	.92	1.93	.93
Item 9	1.56	.74	1.98	.89	1.89	.99
Item 10	1.74	.88	2.00	.94	2.00	.91
Item 12	2.00	.81	2.15	.87	2.11	1.00
Item 14	1.71	.81	1.94	.90	1.93	1.03
Item 16	1.78	.87	1.97	.93	2.14	1.09
Item 17	1.94	.86	2.05	.86	2.01	.99
Item 19	1.98	.90	2.30	.98	2.34	1.19
Total score	18.51	6.06	21.40	7.07	20.95	7.78

Item 1: Interested; Item 3: Excited; Item 5: Strong; Item 9: Enthusiastic; Item 10: Proud; Item 12: Alert; Item 14: Inspired; Item 16: Determined; Item 17: Attentive; Item 19: Active

**Table 6:** Descriptive statistics for each item and the total score on the PANAS-N

	Depression (*n* = 238)		Anxiety (*n* = 284)		Adjustment disorder (*n* = 74)	
	M	SD	M	SD	M	SD
Item 2	3.47	1.04	3.61	1.06	3.68	1.09
Item 4	3.03	1.15	2.93	1.12	2.96	1.08
Item 6	2.87	1.39	2.63	1.27	2.78	1.39
Item 7	2.70	1.29	2.92	1.31	2.62	1.29
Item 8	2.28	1.08	2.19	1.09	2.36	1.14
Item 11	2.90	1.20	2.80	1.20	2.72	1.14
Item 13	2.24	1.27	2.41	1.32	2.32	1.32
Item 15	3.35	1.12	3.57	1.12	3.30	1.08
Item 18	3.28	1.12	3.43	1.11	3.24	1.02
Item 20	2.81	1.39	3.11	1.34	2.68	1.34
Total score	28.92	8.06	29.31	8.32	28.66	7.75

Item 2: Distressed; Item 4: Upset; Item 6: Guilty; Item 7: Scared; Item 8: Hostile; Item 11: Irritable; Item 13: Ashamed; Item 15: Nervous; Item 18: Jittery; Item 20: Afraid

On the PANAS-P, the results of one-way ANOVAs yielded statistically significant differences based on civil status, *F*(3, 591) = 3.05, *p* < .05, diagnostic category, *F*(2, 592) = 12.22, *p* < .001, principal diagnosis, *F*(10, 584) = 5.56, *p* < .001, and number of comorbid diagnoses, *F*(3, 590) = 2.92, *p* < .05. Sidak’s post hoc tests showed that patients in the category of depressive disorders had significantly lower scores on PA than those in the categories of anxiety (*p* < .001) and adjustment disorders (*p* < .05). Additionally, the results of Sidak’s post hoc tests showed that patients with MDD as the principal diagnosis had significantly lower scores on PA than patients with GAD (*p* < .01), AG (*p* < .01) and OCD (*p* < .001). On the PANAS-N, no significant differences were found on any of the sociodemographic or clinical variables, except for the number of comorbid diagnoses, *F*(3, 590) = 9.07, *p* < .001). Sidak’s post hoc tests showed that patients with one (*p* < .01), two (*p* < .05) or three (*p* < .01) comorbid disorders had significantly higher scores on NA, compared to patients with no comorbid diagnoses.

#### Sensitivity to change

To examine potential floor and ceiling effects for the PANAS-P and PANAS-N scores, the frequencies and percentages of minimum (10) and maximum (50) scores on the pretest were tabulated for each sample, using the participants in the treatment and control groups. As Table 7 shows, floor and ceiling effects can be ruled out because the percentage of minimum and maximum scores was less than 15% in all three studies.

**Table 7:** Frequency (and %) of minimum (10) and maximum (50) scores on the pretest in the three studies

Sample	*N*	Minimum (10)	Maximum (50)
S1:			
PANAS-P	207	4(1.9%)	0(0%)
PANAS-N	207	0(0%)	1(0.5%)
S2:			
PANAS-P	35	2(5.7%)	0(0%)
PANAS-N	35	0(0%)	0(0%)
S3:			
PANAS-P	125	4(3.2%)	0(0%)
PANAS-N	125	0(0%)	0(0%)

*S* sample, *N* sample size, *PANAS-P* Positive and Negative Affect Schedule - Positive Affect, *PANAS-N* Positive and Negative Affect Schedule - Negative Affect

In addition, means and standard deviations for the pretest and posttest were calculated for the treatment groups in each sample. The statistical significance of the pretest-posttest change scores was assessed by applying *t*-tests. As Table 8 shows, statistically significant pretest-posttest differences were found in the three studies for both PANAS-P and PANAS-N scores. Clinical significance was assessed by means of the effect size index ‘standardized mean change index’ (*d*). Following Rubio-Aparicio et al.’s (2017) results, with the exception of the PANAS-P scores in Sample 3, all effect sizes were moderate to large (all over 0.74).

**Table 8:** Descriptive and inferential results from the three samples for the PANAS on the pre-posttest scores

Sample	*N*	Pretest		Posttest		*t*	*d*[95% CI]
		Mean	*SD*	Mean	*SD*		
S1:							
PANAS-P	136	20.51	6.90	26.42	9.10	8.62***	0.74[0.54; 0.93]
PANAS-N	136	31.24	8.17	21.01	8.28	14.20***	1.21[0.99; 1.43]
S2:							
PANAS-P	16	20.00	8.97	32.00	8.45	5.74***	1.36[0.63; 2.09]
PANAS-N	16	30.44	8.14	20.69	6.60	4.21***	1.00[0.37; 1.63]
S3:							
PANAS-P	105	19.12	6.16	23.64	8.45	6.32***	0.61[0.40; 0.82]
PANAS-N	105	27.99	8.47	21.75	8.65	8.04***	0.78[0.56; 1.00]

*S* sample, *N* sample size, *SD* standard deviation; T: t statistic for testing the pretest-posttest mean difference; *** *p* < .001. d: standardized mean change index (95%CI)

## Discussion

The present study was designed to evaluate the psychometric properties of the PANAS scale in a Spanish clinical sample with anxiety, depressive, and adjustment disorders. In addition, its objective was to validate the scale administered in an online format, thus contributing to the use of evidence-based assessment. At the same time, the current study investigated the factorial validity of the Spanish version of the PANAS using confirmatory factor analyses.

Preliminary analyses showed no statistically significant differences based on gender, education level, or marital status. However, statistically significant differences were found depending on the principal diagnosis, showing that patients with depression had lower levels of PA than patients with anxiety and adjustment disorders. No differences in NA were found based on the principal diagnosis. These results are consistent with the literature, indicating that low levels of PA predict the onset of depression [12], reduce positive emotions, and increase the severity of the depressive symptoms [67]. In fact, the literature shows that depressive symptoms often involve low levels of positive emotions, and that low levels of positive affect are more strongly linked to depression than to other emotional disorders [63, 68, 69].

Furthermore, regarding the number of comorbid disorders, 35% of the total sample presented at least one comorbid anxiety or depressive disorder. The results reveal that patients with more comorbid diagnoses had significantly higher NA and lower PA than patients with fewer comorbid disorders, which coincides with evidence showing that severity is strongly related to comorbidity [70]. For reliability, it is generally accepted that alpha coefficients must be over .70 for exploratory research, over .80 for general research purposes, and over .90 when the test is used for clinical decisions [71]. In addition, Cicchetti (1994) [72] suggested the following guidelines for assessing the clinical relevance of alpha coefficients: unacceptable for coefficients lower than .7, fair for the .7–.8 range, good for the .8–.9 range, and excellent for values over .9. Therefore, according to these criteria, both PANAS-P and PANAS-N exhibited good to excellent internal consistency reliability (alphas of .91 and .87, respectively).

Regarding construct validity, convergent and divergent validity were demonstrated by correlations between PANAS-P and PANAS-N and depression, anxiety, and personality measures. Overall, significant correlations were found between positive and negative affect and all the measures. Convergent and discriminant validity were supported because NA scores were strongly correlated with symptoms of depression (measured by the BDI-II and PHQ-9) and anxiety (measured by the BAI and OASIS), whereas PA showed strong inverse correlations with depression measures and small to medium correlations with anxiety measures. Finally, as expected, PANAS-P correlated significantly with NEO-FFI-E and negatively with NEO-FFI-N. At the same time, PANAS-N correlated significantly with NEO-FFI-N, and inversely with NEO-FFI-E. Although the small correlation found between PANAS-N and NEO-FFI-E might stand out, this result is theoretically expected due to the commonalities shown between the affectivity and temperament dimensions of neuroticism and extraversion. The presence of high levels of neuroticism has been related to experiences of NA, whereas PA has traditionally been more related to the extraversion dimension [62, 63].

This study also examined the latent structure of the PANAS, and Confirmatory Factor Analysis was performed. The confirmatory factor analysis yielded a latent structure of two independent factors, coinciding with previous validations of the instrument and with the theoretical framework of positive and negative affect that conceptualizes them as two independent temperamental dimensions [9, 10, 15].

Regarding sensitivity to change, this study also analyzed the significance of the improvements from pre- to post-treatment on the PANAS scores. The results showed statistically significant pretest-posttest differences in the three studies for both PANAS-P and PANAS-N, with moderate to large effect sizes, suggesting that the scale is able to detect changes in affectivity and, therefore, can be used to examine the impact of an intervention.

Overall, the results obtained in this study show adequate psychometric properties for the online version of the PANAS as a measure for the assessment of positive and negative affect in a Spanish clinical population. These results reinforce the importance of validating online assessment instruments, given the current proliferation of Internet-based psychological treatments.

The present study has several strengths. First, to our knowledge, this is the first study to evaluate the psychometric properties of the PANAS in a Spanish clinical population. Second, this is the first validation of this instrument in an online format. The literature presents considerable evidence about the efficacy of Internet-based interventions for emotional disorders [73, 74]. Recently in our country, studies have been carried out that also guarantee their efficacy [75–77], and so it is important to have validated online assessment tools that can be used to assess PA and NA (two core symptoms) in research and clinical settings. In this regard, the present study provides empirical evidence about the usefulness of the instrument in distinguishing between PA and NA with a digital version of the questionnaire. Furthermore, online assessment also has advantages in clinical practice and routine care, where there are always time limitations. In this way, it is possible to reach more people in less time than with paper and pencil evaluations. Research results encourage the use of online questionnaires because they offer many advantages over traditional data collection methods [78–80]. For instance, missing data can be handled in a more straightforward way, and scoring is easy and immediate [78]. In addition, online assessment allows users to receive feedback about their progress [81]. Finally, the high diagnostic heterogeneity in the present study (i.e. individuals with anxiety, depressive, and adjustment disorders) helps to increase the generalizability of the results.

### Limitations

This study has several limitations that should be mentioned. First, it was not possible to calculate sensitivity to change with the whole sample because post-treatment scores were not available for all the participants in this study. Second, the validation was limited to participants with psychological disorders. Therefore, we were not able to obtain cutoff scores on the PANAS. Finally, we did not evaluate the test-retest reliability of the PANAS. Literature has shown that there is a wide variety of administration intervals used in test-retest reliability [82]. However, the length of the test-retest interval can affect patient’s responses and should be sufficiently long to ensure that patients do not remember their previous answer, though sufficiently short to ensure that clinical change has not occurred over time [83]. The most common period that has been stablished as appropriate is 1 or 2 weeks [83]. In this study, due to the long time interval between the two measurement times (pretest-posttest) in each clinical samples (sample 1: 16 weeks; sample 2: 7 weeks; sample 3: 4 weeks) and the knowledge that results could have been contaminated over time, we decided not to evaluate the test-retest reliability.

## Conclusion

In conclusion, the results of this study support the adequacy of the PANAS applied online in Spanish clinical samples suffering from different mental disorders (i.e. anxious, depressive, and adjustment disorders). Overall, the present study provides a psychometrically-validated online measure to assess the structure of affect, and it supports the application of the PANAS in clinical settings. Future research should analyze the validity and reliability of the PANAS in more severe patients (e.g., bipolar or psychotic disorders). This online validation can have a direct impact on evidence-based assessment, facilitating access to appropriate instruments for evaluating affectivity in mental disorders.

## Supplementary information

**Additional file 1.** PANAS (Spanish version).

## Data Availability

The data underlying this study have been uploaded to Zenodo and are accessible using the following DOI: 10.5281/zenodo.1477084

## Abbreviations

AG: Agoraphobia
ANOVA: Analysis of variance
Anxiety NOS: Anxiety Not Otherwise Specified
BAI: Beck Anxiety Inventory
BDI-II: Beck Depression Inventory
CFA: Confirmatory Factor Analysis
CFI: Comparative Fit Index
DD: Dysthymic Disorder
Depression NOS: Depression Not Otherwise Specified
DSM-IV: Diagnostic and Statistical Manual of mental disorders, fourth text
GAD: Generalized Anxiety Disorder
ICD-10: International Classification of Diseases, tenth edition
MDD: Major Depression Disorder
MINI: Mini International Neuropsychiatric Interview
NA: Negative Affect
NEO-FFI: NEO Five Factor Inventory
NEO-FFI-E: NEO Five-Factor Inventory – Extraversion
NEO-FFI-N: Neo Five-Factor Inventory – Neuroticism
OASIS: Overall Anxiety Severity and Impairment Scale
OCD: Obsessive-Compulsive Disorder
PA: Positive Affect
PANAS: Positive and Negative Affect Schedule
PANAS-N: Positive and Negative Affect Schedule - Negative Affect
PANAS-P: Positive and Negative Affect Schedule - Positive Affect
PD: Panic Disorder
PHQ-9: Patient Health Questionnaire
RMSEA: Root Mean Square Error of Approximation
SAD: Social Anxiety Disorder
SD: Standard Deviation
SRMR: Standardized Root Mean Residual
WLC: Waitlist control group
WLSMV: Weighted Least Square Mean and Variance corrected

## References

[1] Gray EK, Watson D. Assessing positive and negative affect via self-report. Handbook of emotion elicitation and assessment; 2007.

[2] Rosenberg Erika L. (1998). Levels of Analysis and the Organization of Affect. Review of General Psychology.

[3] Gross J, Sutton SK, Ketelaar T (1998). Relations between affect and personality: support for the affect-level and affective-reactivity views. Clin Soc Psychol Bull.

[4] Parkinson B, Totterdell P, Briner R, Reynolds S (1996). Changing moods: the psychology of mood and mood regulation.

[5] Lucas RE, Diener E, Larsen RJ (2003). Measuring positive emotions. Positive psychological assessment: a handbook of models and measures.

[6] Russell JA (1980). A Circumplex model of affect. J Pers Soc Psychol.

[7] Zevon Michael A., Tellegen Auke (1982). The structure of mood change: An idiographic/nomothetic analysis. Journal of Personality and Social Psychology.

[8] Watson David, Clark Lee Anna, Tellegen Auke (1984). Cross-cultural convergence in the structure of mood: A Japanese replication and a comparison with U.S. findings. Journal of Personality and Social Psychology.

[9] Bradburn NM (1969). The structure of psychological well-being.

[10] Watson David, Tellegen Auke (1985). Toward a consensual structure of mood. Psychological Bulletin.

[11] Watson D, Clark LA, Tellegen A (1988). Development and validation of brief measures of positive and negative affect: the PANAS scales. J Pers Soc Psychol.

[12] Snyder CR, Lopez SJ. Oxford handbook of positive psychology. Oxford library of psychology; 2009.

[13] Fredrickson Barbara L. (2001). The role of positive emotions in positive psychology: The broaden-and-build theory of positive emotions. American Psychologist.

[14] Tellegen A. Structures of mood and personality and their relevance to assessing anxiety, with an emphasis on self-report. In A. H. Tuma & J. D. Maser (Eds.), Anxiety and the anxiety disorders (p. 681–706). Lawrence Erlbaum Associates, Inc; 1985.

[15] Watson D, Clark LA (1992). On traits and temperament: general and specific factors of emotional experience and their relation to the five-factor model. J Pers.

[16] Terracciano A, McCrae RR, Costa PT (2003). J. Factorial and construct validity of the Italian positive and negative affect schedule (PANAS). Eur J Psychol Assess.

[17] Gaudreau P, Sanchez X, Blondin J-P (2006). Positive and negative affective states in a performance-related setting. Eur J Psychol Assess.

[18] Gyollai A, Simor P, Koteles F, Demetrovics Z (2011). Psychometric properties of the Hungarian version of the original and the short form of the positive and negative affect schedule (PANAS). Neuropsychopharmacol Hung.

[19] Gençöz T. Positive and Negative Affect Schedule: A study of validity and reliability. Türk Psikoloji Dergisi. 2000;15(46):19-28.

[20] Robles R, Páez F (2003). Estudio sobre la traducción al español y las propiedades psicométricas de las escalas de afecto positivo y negativo (panas). Salud Ment.

[21] Lim Y-J, Yu B-H, Kim D-K, Kim J-H (2010). The positive and negative affect schedule: psychometric properties of the Korean version. Psychiatry Investig.

[22] Moriondo M, De Palma P, Medrano LA, Murillo P (2012). Adaptación de la escala de afectividad positiva y negativa (PANAS) a la población de adultos de la ciudad de córdoba: Análisis psicométricos preliminares. Univ Psychol.

[23] Pires P, Filgueiras A, Ribas R, Santana C (2013). Positive and negative affect schedule: psychometric properties for the Brazilian Portuguese version. Span J Psychol.

[24] Merz EL, Malcarne VL, Roesch SC, Ko CM, Emerson M, Roma VG (2013). Psychometric properties of positive and negative affect schedule (PANAS) original and short forms in an African American community sample. J Affect Disord.

[25] Pandey R, Srivastava N. Psychometric evaluation of a hindi version of positive-negative affect schedule. Ind Psychiatry J. 2008;17(1):49.

[26] Akhter N (2017). Urdu translation and validation of shorter version of positive affect and negative affect schedule (PANAS) on Pakistani bank employees. J Pak Med Assoc.

[27] Sandín B, Chorot P, Lostao L, Joiner TE, Santed MA, Valiente RM (1999). Escalas PANAS de afecto positivo y negativo: Validacion factorial y convergencia transcultural. Psicothema.

[28] Leue A, Beauducel A (2011). The PANAS structure revisited: on the validity of a Bifactor model in community and forensic samples. Psychol Assess.

[29] Serafini K, Malin-Mayor B, Nich C, Hunkele K, Carroll KM (2016). Psychometric properties of the positive and negative affect schedule (PANAS) in a heterogeneous sample of substance users. Am J Drug Alcohol Abuse.

[30] Estévez-López F, Pulido-Martos M, Armitage CJ, Wearden A, Álvarez-Gallardo IC, Arrayás-Grajera MJ (2016). Factor structure of the positive and negative affect schedule (PANAS) in adult women with fibromyalgia from southern Spain: the al-Ándalus project. Peer J.

[31] Alfonsson Sven, Maathz Pernilla, Hursti Timo (2014). Interformat Reliability of Digital Psychiatric Self-Report Questionnaires: A Systematic Review. Journal of Medical Internet Research.

[32] Seib-Pfeifer L-E, Pugnaghi G, Beauducel A, Leue A (2017). On the replication of factor structures of the positive and negative affect schedule (PANAS). Pers Individ Dif.

[33] van Ballegooijen W, Riper H, Cuijpers P, van Oppen P, Smit JH (2016). Validation of online psychometric instruments for common mental health disorders: a systematic review. BMC Psychiatry.

[34] Hunsley J, Mash EJ (2007). Evidence-based assessment. Annu Rev Clin Psychol.

[35] Díaz-García A, González-Robles A, Fernández-Álvarez J, García-Palacios A, Baños RM, Botella C (2017). Efficacy of a transdiagnostic internet-based treatment for emotional disorders with a specific component to address positive affect: study protocol for a randomized controlled trial. BMC Psychiatry.

[36] Rachyla I, Pérez-Ara M, Molés M, Campos D, Mira A, Botella C (2018). An internet-based intervention for adjustment disorder (TAO): study protocol for a randomized controlled trial. BMC Psychiatry.

[37] Castro A, García-Palacios A, García-Campayo J, Mayoral F, Botella C, García-Herrera JM (2015). Efficacy of low-intensity psychological intervention applied by ICTs for the treatment of depression in primary care: a controlled trial. BMC Psychiatry.

[38] Sheehan DV, Lecrubier Y, Sheehan KH, Amorim P, Janavs J, Weiller E (1998). The Mini-International Neuropsychiatric Interview (M.I.N.I.): the development and validation of a structured diagnostic psychiatric interview for DSM-IV and ICD-10. J Clin Psychiatry.

[39] Lecrubier Y, Sheehan DV, Weiller E, Amorim P, Bonora I, Sheehan KH (1997). The MINI international neuropsychiatric interview (MINI). A short diagnostic structured interview: reliability and validity according to the CIDI. Eur Psychiatry.

[40] Ferrando L, Bobes J, Gibert J (2000). MINI. Mini International Neuropsychiatric Interview. Versión en Español 5.0.0 DSM-IV. Instrumentos Detección y Orientación Diagnóstica.

[41] Beck AT, Steer RA, Brown GK (1996). Beck depression inventory.

[42] Sanz J, Navarro ME, Vázquez C (2003). Adaptación española del Inventario para la Depresión de Beck-II (BDI-II): propiedades psicométricas en estudiantes universitarios. Análisis y Modif Conduct.

[43] Sanz J, Perdigón AL, Vázquez C (2003). Adaptación española del Inventario para la Depresión de Beck-II (BDI-II): 2. Propiedades psicométricas en población general. Clin y Salud.

[44] Sanz J, García-Vera MP, Espinosa R, Fortún M, Vázquez C (2005). Adaptación española del Inventario para la Depresión de Beck-II (BDI-II): 3. Propiedades psicométricas en pacientes con trastornos psicológicos. Clínica y Salud.

[45] Beck AT, Brown G, Epstein N, Steer RA (1988). An inventory for measuring clinical anxiety: psychometrical properties. J Consult ans Clincal Psychol.

[46] Sanz J, Navarro ME (2003). Propiedades psicométricas de una versión española del Inventario de Ansiedad de Beck (BAI) en estudiantes universitarios. Ansiedad y Estrés.

[47] Magán I, Sanz J, García-Vera MP (2008). Psychometric properties of a Spanish version of the Beck anxiety inventory (BAI) in general population. Span J Psychol.

[48] Norman SB, Campbell-Sills L, Hitchcock CA, Sullivan S, Rochlin A, Wilkins KC (2011). Psychometrics of a brief measure of anxiety to detect severity and impairment: the overall anxiety severity and impairment scale (OASIS). J Psychiatr Res.

[49] González-Robles A, Mira A, Miguel C, Molinari G, Díaz-García A, García-Palacios A (2018). A brief online transdiagnostic measure: Psychometric properties of the Overall Anxiety Severity and Impairment Scale (OASIS) among Spanish patients with emotional disorders. PLoS One.

[50] Kroenke K, Spitzer RL, Williams JBW (2001). The PHQ-9 validity of a brief depression severity measure. J Gen Intern Med banner.

[51] Muñoz-Navarro R, Cano-Vindel A, Medrano LA, Schmitz F, Ruiz-Rodríguez P, Abellán-Maeso C (2017). Utility of the PHQ-9 to identify major depressive disorder in adult patients in Spanish primary care centres. BMC Psychiatry.

[52] Costa PT, McCrae RR (1992). Normal personality assessment in clinical practice: the NEO personality inventory. Psychol Assess.

[53] Aluja A (2005). García O, Rossier J, García LF. Comparison of the NEO-FFI, the NEO-FFI-R and an alternative short version of the NEO-PI-R (NEO-60) in Swiss and Spanish samples. Pers Individ Dif.

[54] DeVellis Robert F. (2006). Classical Test Theory. Medical Care.

[55] Caycho-Rodriguez T, Ventura-Leon J, Garcia-Cadena CH, Tomas JM, Dominguez-Vergara J, Daniel L (2018). Psychometric evidence of a brief measure of resilience in non-institutionalized Peruvian older adults. Psychosoc Interv.

[56] Finney S, DiStefano C (2013). Non-normal and categorical data in structural equation modeling. Struct Equ Model A Second course.

[57] Hoyle RH, Gottfredson NC (2015). Sample size considerations in prevention research applications of multilevel modeling and structural equation modeling. Prev Sci.

[58] Tanaka, JS. Multifaceted conceptions of fit in structural equation models. In Bollen KA, Long JS (Eds.) Testing structural equation models. 1993:10-40.

[59] Marsh HW, Hau K-T, Wen Z (2004). In search of Golden rule: comment on hypothesis testing approaches to setting cutoff value for fit indexes and danger in overgeneralizing Hu and Bentler’s (1999) finding. Struct Equ Model.

[60] Muthén LK, Muthén BO (1998). Statistical Analysis With Latent Variables User’s Guide.

[61] Cohen J (1988). Statistical power analysis for the behavioral sciences.

[62] Barlow DH, Sauer-Zavala S, Carl JR, Bullis JR, Ellard KK (2014). The nature, diagnosis, and treatment of neuroticism: Back to the future. Clin Psychol Sci.

[63] Brown TA, Barlow DH (2009). A proposal for a dimensional classification system based on the shared features of the DSM-IV anxiety and mood disorders: implications for assessment and treatment. Psychol Assess.

[64] McHorney CA, Tarlov AR (1995). Individual-patient monitoring in clinical practice: are available health status surveys adequate?. Qual Life Res.

[65] Morris SB, DeShon RP (2002). Combining effect size estimates in meta-analysis with repeated measures and independent-groups designs. Psychol Methods.

[66] Rubio-Aparicio M, Marín-Martínez F, Sánchez-Meca J, López-López JA (2018). A methodological review of meta-analyses of the effectiveness of clinical psychology treatments. Behav Res Methods.

[67] Gilbert KE, Nolen-Hoeksema S, Gruber J (2013). Positive emotion dysregulation across mood disorders: how amplifying versus dampening predicts emotional reactivity and illness course. Behav Res Ther.

[68] Barlow D, Allen LB, Choate ML (2004). Toward a unified treatment for emotional disorders. Behav Ther.

[69] Watson D, Naragon-Gainey K (2010). On the specificity of positive emotional dysfunction in psychopathology: evidence from the mood and anxiety disorders and schizophrenia/schizotypy. Clin Psychol Rev.

[70] Kessler RC, Chiu WT, Demler O, Merikangas KR, Walters EE (2005). Prevalence, severity, and comorbidity of 12-month DSM-IV disorders in the National Comorbidity Survey Replication. Arch Gen Psychiatry.

[71] Nunnally J, Bernstein I (1994). Psychometric Theory 3rd edition.

[72] Cicchetti DV (1994). Guidelines, criteria, and rules of thumb for evaluating normed and standardized assessment instruments in psychology. Psychol Assess.

[73] Andersson G, Cuijpers P (2009). Internet-based and other computerized psychological treatments for adult depression: a meta-analysis. Cogn Behav Ther.

[74] Andrews G, Cuijpers P, Craske MG, McEvoy P, Titov N (2010). Computer Therapy for the Anxiety and Depressive Disorders Is Effective, Acceptable and Practical Health Care: A Meta-Analysis. PLoS One.

[75] Mira A, Bretón-López J, García-Palacios A, Quero S, Baños RM, Botella C (2017). An internet-based program for depressive symptoms using human and automated support: a randomized controlled trial. Neuropsychiatr Dis Treat.

[76] Montero-Marín J, Araya R, Pérez-Yus MC, Mayoral F, Gili M, Botella C (2016). An Internet-Based Intervention for Depression in Primary Care in Spain: A Randomized Controlled Trial. J Med Internet Res.

[77] Romero-Sanchiz Pablo, Nogueira-Arjona Raquel, García-Ruiz Antonio, Luciano Juan V., García Campayo Javier, Gili Margalida, Botella Cristina, Baños Rosa, Castro Adoración, López-Del-Hoyo Yolanda, Pérez Ara Mª Ángeles, Modrego-Alarcón Marta, Mayoral Cleríes Fermín (2017). Economic evaluation of a guided and unguided internet-based CBT intervention for major depression: Results from a multi-center, three-armed randomized controlled trial conducted in primary care. PLOS ONE.

[78] Carlbring P, Brunt S, Bohman S, Austin D, Richards J, Öst L-G (2007). Internet vs. paper and pencil administration of questionnaires commonly used in panic/agoraphobia research. Comput Human Behav.

[79] Hedman E, Ljótsson B, Rück C, Furmark T, Carlbring P, Lindefors N (2010). Internet administration of self-report measures commonly used in research on social anxiety disorder: a psychometric evaluation. Comput Human Behav.

[80] Vallejo MA, Jordán CM, Díaz MI, Comeche MI, Ortega J (2007). Psychological assessment via the internet: a reliability and validity study of online (vs paper-and-pencil) versions of the general health Questionnaire-28 (GHQ-28) and the symptoms check-List-90-revised (SCL-90-R). J Med Internet Res.

[81] Barak A, English N (2002). Prospects and limitations of psychological testing on the internet. J Technol Hum Serv.

[82] Quadri N, Wild D, Skerritt B, Muehlhausen W, O'Donohoe P (2013). A literature review of the variance in interval length between administrations for assessment of test retest reliability and equivalence of pro measures. Value Health.

[83] Terwee CB, Bot SDM, de Boer MR, van der Windt DAWM, Knol DL, Dekker J (2007). Quality criteria were proposed for measurement properties of health status questionnaires. J Clin Epidemiol.

