# Supplementary material for: Positive and Negative Affect Schedule (PANAS): psychometric properties of the online Spanish version in a clinical sample with emotional disorders
Source: BMC Psychiatry. 2020 Feb 10;20:56. doi: 10.1186/s12888-020-2472-1 (PMC7008531; doi:10.1186/s12888-020-2472-1)
Supplement: Supplementary file 1 — Additional file 1. PANAS (Spanish version). [file 12888_2020_2472_MOESM1_ESM.doc]

INSTRUCCIONES: A continuación, se indican una serie de palabras que describen sentimientos y emociones. Lee cada una de ellas y contesta hasta qué punto sueles sentirte **HABITUALMENTE** de la forma que indica cada expresión.

**Generalmente** me siento:

|  | **Nada o casi nada** | **Un poco** | **Bastante** | **Mucho** | **Muchísimo** |
| --- | --- | --- | --- | --- | --- |
| 1. Interesado/a por las cosas. | 1 | 2 | 3 | 4 | 5 |
| 1. Estresado/a, tenso/a. | 1 | 2 | 3 | 4 | 5 |
| 1. Emocionado/a, ilusionado/a. | 1 | 2 | 3 | 4 | 5 |
| 1. Disgustado/a, molesto/a. | 1 | 2 | 3 | 4 | 5 |
| 1. Con energía, con vitalidad. | 1 | 2 | 3 | 4 | 5 |
| 1. Culpable. | 1 | 2 | 3 | 4 | 5 |
| 1. Asustado/a. | 1 | 2 | 3 | 4 | 5 |
| 1. Hostil. | 1 | 2 | 3 | 4 | 5 |
| 1. Entusiasmado/a. | 1 | 2 | 3 | 4 | 5 |
| 1. Orgulloso/a (de algo), satisfecho/a conmigo mismo/a. | 1 | 2 | 3 | 4 | 5 |
| 1. Irritable, malhumorado/a. | 1 | 2 | 3 | 4 | 5 |
| 1. Despejado/a, despierto/a. | 1 | 2 | 3 | 4 | 5 |
| 1. Avergonzado/a. | 1 | 2 | 3 | 4 | 5 |
| 1. Inspirado/a. | 1 | 2 | 3 | 4 | 5 |
| 1. Nervioso/a. | 1 | 2 | 3 | 4 | 5 |
| 1. Decidido/a. | 1 | 2 | 3 | 4 | 5 |
| 1. Atento/a (a las cosas), concentrado/a. | 1 | 2 | 3 | 4 | 5 |
| 1. Intranquilo/a, inquieto/a. | 1 | 2 | 3 | 4 | 5 |
| 1. Activo/a. | 1 | 2 | 3 | 4 | 5 |
| 1. Con miedo, miedoso/a. | 1 | 2 | 3 | 4 | 5 |

INSTRUCTIONS: This scale consists of a number of words that describe different feelings and emotions. Read each item and then mark the appropriate answer in the space next to that word. Indicate to what extent you GENERALLY feel this way, that is, how you feel on the average.

|  | **Very slightly or not at all** | **A little** | **Moderately** | **Quite a bit** | **Extremely** |
| --- | --- | --- | --- | --- | --- |
| 1. Interested. | 1 | 2 | 3 | 4 | 5 |
| 1. Distressed. | 1 | 2 | 3 | 4 | 5 |
| 1. Excited. | 1 | 2 | 3 | 4 | 5 |
| 1. Upset. | 1 | 2 | 3 | 4 | 5 |
| 1. Strong. | 1 | 2 | 3 | 4 | 5 |
| 1. Guilty. | 1 | 2 | 3 | 4 | 5 |
| 1. Scared. | 1 | 2 | 3 | 4 | 5 |
| 1. Hostile. | 1 | 2 | 3 | 4 | 5 |
| 1. Enthusiastic. | 1 | 2 | 3 | 4 | 5 |
| 1. Proud. | 1 | 2 | 3 | 4 | 5 |
| 1. Irritable. | 1 | 2 | 3 | 4 | 5 |
| 1. Alert. | 1 | 2 | 3 | 4 | 5 |
| 1. Ashamed. | 1 | 2 | 3 | 4 | 5 |
| 1. Inspired. | 1 | 2 | 3 | 4 | 5 |
| 1. Nervous. | 1 | 2 | 3 | 4 | 5 |
| 1. Determined. | 1 | 2 | 3 | 4 | 5 |
| 1. Attentive. | 1 | 2 | 3 | 4 | 5 |
| 1. Jittery. | 1 | 2 | 3 | 4 | 5 |
| 1. Active. | 1 | 2 | 3 | 4 | 5 |
| 1. Afraid. | 1 | 2 | 3 | 4 | 5 |
